# Oral manifestations of gastrointestinal disorders

**DOI:** 10.4317/jced.54008

**Published:** 2017-10-01

**Authors:** Martin Jajam, Patricia Bozzolo, Sven Niklander

**Affiliations:** 1 Facultad de Odontología, Universidad Andres Bello, Chile

## Abstract

**Background:**

A considerable number of gastrointestinal disorders (GIDs) of varied nature (inflammatory, infectious, genetic and other etiology) may produce alterations in the hard and soft oral tissues. Among these are Crohn’s disease, ulcerative colitis, celiac and gastroesophageal reflux disease.

**Material and Methods:**

Article search was done using the National library of medicine (PubMed) database using different search terms and analyzed according to their importance.

**Results:**

A large variety of GIDs can give rise to oral lesions, including: RAS like ulceration, mucosal tags, cobblestoning, mucogingivitis, labial and facial swelling, pyostomatitis vegetans, disgeusia and dental abnormalities, among others. Although in most cases the gastrointestinal signs and symptoms highlight in the clinical picture, a considerable percentage of these patients are affected by oral manifestations before the onset of gastrointestinal symptoms. This lesions can cause significant functional and aesthetics damages as well deteriorate the patient quality of life.

**Conclusions:**

Although the frequency of oral manifestations is variable across GIDs and in most cases is non-specific, these alterations may precede the underlying disease and therefore can facilitate an opportune diagnosis.

** Key words:**Gastrointestinal disorders, oral lesions, oral mucosal disorders.

## Introduction

It is well known that a considerable number of systemic diseases can affect the oral cavity. Among these are the gastrointestinal disorders (GIDs), which have a high worldwide prevalence and a growing incidence. Although gastrointestinal signs and symptoms are predominant, oral manifestations may occur and even herald the onset of the underlying GID.

## Material and Methods

The search of articles was done using the National library of medicine (PubMed) database. The following search terms were used: Gastrointestinal diseases, Gastrointestinal pathology, Inflammatory bowel diseases, Crohn´s disease, Ulcerative colitis, Gardner´s syndrome, Peutz-Jeghers syndrome, Celiac disease, Coeliac disease, Celiac sprue, Peptic Ulcer, Helicobacter pylori gastritis, Gas-troesophageal reflux disease, Pernicious anemia, Plummer Vinson syndrome, Patterson-Kelly syndrome and Hiatal Hernia. Each of above terms were combined with the following words: AND oral cavity, AND oral mucosa, AND buccal mucosa, AND orofacial region, AND oral manifestation, AND mouth. The studies included were those articles in English that had the search words in their title, abstract or text. Those articles that did not have information about the topics of the diseases treated in this review or with clear mistakes in their methodology were excluded. Although systematic reviews were the study design of predilection, case report and case series studies were also included because of the rarity of some diseases.

## Results

As shown in  Table 1, many GIDs can give rise to different oral lesions. In this review we will focus in the most frequent gastrointestinal disorders.

**Table 1:**
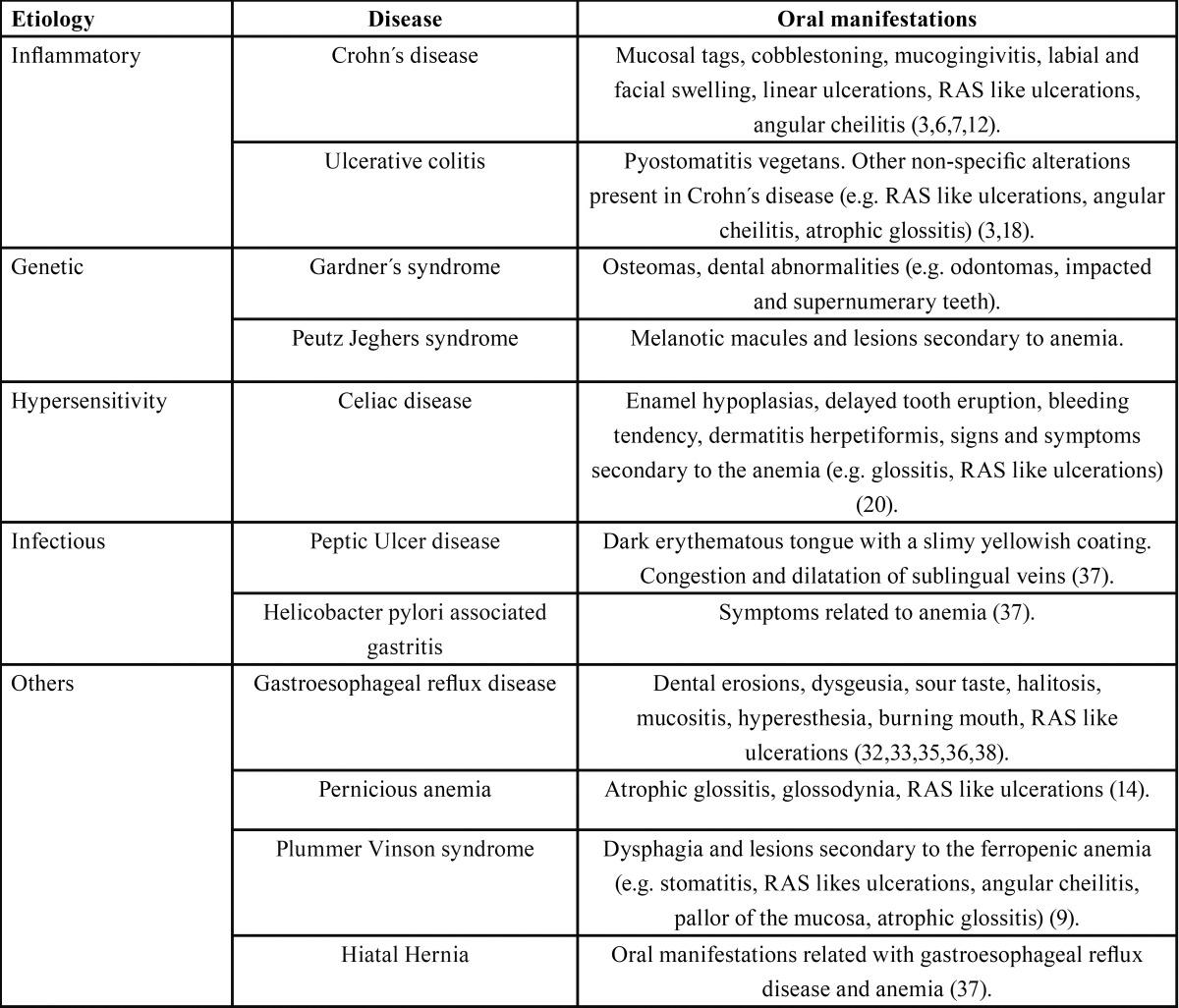
Oral manifestations of gastrointestinal disorders.

-Inflammatory bowel diseases 

The term inflammatory bowel disease (IBD) involves a group of chronic inflammatory disorders of not well known etiology that affects different portions of the gastrointestinal tract, mainly the bowels (1). The two main forms of IBD are Crohn’s disease (CD) and Ulcerative Colitis (UC) (2). The signs and symptoms are related to the damage in the bowel, but in some cases the patient can exhibit extra intestinal manifestations on the oral cavity, even before to the outbreak in the bowels (2-4).

-Crohn’s disease

Patients with Crohn´s disease develop chronic inflammation and non-caseating granulomas in different parts of the gastrointestinal tract, especially in the distal ileum and colon (5). The most common signs and symptoms include diarrhea and abdominal pain, but some patients could experience extra-intestinal manifestations of the disease, involving eyes, joints, skin and mouth (6).

Epidemiology

CD usually affects males in their third decade of life; however, it can appear in a wide range of ages including young children’s (1). Its incidence varies depending on the age of the group studied, being higher in pediatric patients. Regarding to its prevalence, there are geographical differences, but it has been estimated between 319 and 322 cases per 100,000 habitants (5).

Etiopathogenia

The exact cause and pathogenesis of CD is still not known. It has been postulated that genetically predisposed individuals would present an imbalance or deregulation of their immune response when exposed to different agents, such as environmental (stress, tobacco and diet) and microbiological (bacterial species) agents leading to the pro-inflammatory environment and tissue damage seen in this disease (5).

Oral manifestations

Oral lesions in CD are more frequent in young male patients (3) and their prevalence can range from 20 to 50% (3,7,8). The pre-dominant clinical presentation of the oral lesions includes ulcers, papules and edema, while the most common sites affected are lips, gingiva and the vestibular sulci (9). It has been reported that oral lesions are of help in the diagnosis of systemic Crohn’s disease (10). Patients with active CD have been reported to have a higher degree of oral lesions (7), but apparently, the type of oral lesion has no relationship with the disease activity and type of treatment (3,7,8). Different types of oral lesions can coexist in the same patient, and according to the absence or presence of granulomas formation in the histopathological study, these are classified into specific and non-specific lesions respectively (3,4).

Specific oral lesions:

Labial swelling and fissuring: Consist in a chronic enlargement of the lips with perpendicular fissures, cracks or crusts along the vermilion (3,7,11)

Mucosal tags: Also known as epithelial tags or folds. Consist in white or normal color reticular tags often present in the vestibule and retro-molar region (3,8).

Cobblestoning: The yugal mucosa exhibits normal color plaques separated by mild depressions or fissures, giving the appearance of cobblestones. In some circumstances, these lesions can difficult normal function, such as chewing (3,12).

Mucogingivitis: The gingival tissues may become hyperplasic and granular, not only the free gingiva but also the attached gingi-va, and in certain cases this lesion can be extended up to the mucogingival margin (8).

Linear ulcerations: These lesions are usually located in the buccal sulci and may be accompanied by hyperplastic mucosa at their borders (2,3,8).

Non-specific oral manifestations:

Recurrent aphtous stomatitis (RAS) like ulceration: It is one of the more prevalent lesions among patients with CD, being reported in up to 27% of all CD cases (13). Clinically, RAS like oral ulceration present as recurrent bouts of usually multiple, round or ovoid superficial ulcers that have circumscribed margins surrounded by an erythematous halo, clinically indestenguible from RAS (14). RAS like ulcerations must be differentiated from RAS, because by definition, RAS arises in patients who are otherwise well, which is not the case in these patients. In contrast to the intestinal ulcers, they have no clinical significance (7). It is important to highlight that RAS like lesions are not specific of CD, hence they are present in several disorders (e.g. AIDS, celiac disease, Behcet´s syndrome, anemia) (14).

Angular cheilitis: The commissure and adjacent skin may have recurrent fissures and indurated erythematous plaques not necessarily related with candida infection. According to the study of Lisciandrano *et al.* (7), it is the most prevalent lesion among patients with IBD.

Pyostomatitis vegetans: Although it is described as an oral manifestation in patients with CD (15), it is more frequent in patients with ulcerative colitis and hence will be described in the corresponding section.

Other non-specific oral findings reported in literature: Include submandibular lymphadenopathy, sicca syndrome and hyposialia, dental caries, halitosis, candidiasis, dysphagia, odynophagia, lichen planus, dysgeusia, glossitis, mucosal discoloration, periodontal involvement, perioral erythema with scaling and minor salivary gland enlargement (3,7).

Despite most of the oral manifestations of CD are not severe and their symptoms are mild or absent, some patients may experience facial distortion and disabling pain, originating emotional stress and deteriorating quality of life (9).

Treatment 

The treatment of CD is focused in the control of the underlying intestinal involvement and in some circumstances, of its extra-intestinal manifestations (if they are symptomatic or are causing cosmetic issues). Treatment is usually performed by drug administration (steroids, immunosuppressive and/or biological agents), although some cases are managed surgically (9). Usually oral lesions are well managed with topical steroids, but the use of systemic agents might be necessary for some cases.

-Ulcerative colitis

Ulcerative colitis (UC) is classified together with CD in the group of IBDs, but it has clinical and histopathological features that differentiate it from CD. Firstly, the chronic inflammation of the gastrointestinal tract is limited to the mucosa of the rectum and colon, and only in some rare instances it can spread to upward portions of the digestive tube (e.g. small intestine). Secondly, within the inflammation there is no granuloma formation, which is a main feature of CD. The disease usually progresses with repeatedly periods of remissions and exacerbations, and in severe cases, can affect the entire thickness of the intestinal wall which can cause important bleeding. Digestive signs and symptoms of UC are chronic diarrhea, abdominal pain, weight loss and fatigue (16).

Epidemiology

Ulcerative colitis shows a predilection for males and is twice more frequent than CD (17). Contrary to CD, UC is commonly diagnosed in slightly older patients with a mean age of 32 years (16). The occurrence of UC follows a bimodal pattern, with peaks in the early adulthood and between the sixth and seventh decade of life. Europe is the continent with the highest incidence, with 24.3 new cases per 100,000 person each year (17).

Etiopathogenia

Similarly to CD, it is postulated that the development of UC would be influenced by different factors, including microbiological, genetic and environmental components that would interact between each other triggering the disease (1).

Oral manifestations

Pyostomatitis vegetans (PV): Is the oral counterpart of the Pyoderma gangrenosum and is highly associated with UC, and unlike the majority of the oral lesions, it has been reported to be a specific marker of the disease activity (3,18). PV is cataloged as a chronic muco-cutaneous pathology that consists in the formation of numerous pustules (intra and sub epithelial abscesses) of white-yellowish content with an erythematous and edematous base. These lesions may break or coalesce giving a snail track appearance (18). The patient can experience fever, submandibular adenopathy and pain, symptoms that are highly variable and are not necessarily related with the extension and size of the ulcers (3). In the few cases reported in the literature, the lesions have been found on the tongue, lips, gingiva, tonsillar pillars, buccal mucosa and soft and hard palate (6). As for the etiology, it remains obscure and researches have not been able to attribute a microbiological cause (18).

Others: In some occasions, patients with UC can also be affected by some oral lesions more common in CD, such as RAS like ulcers (reported up to 13% of cases), glossitis, cheilitis, stomatitis, mucosal ulcers and gingival inflammation (3,7,9). These lesions (like in CD) usually arise as a result of the nutritional deficiencies (e.g. iron, folate or B12) secondary to the intestinal involvement and/or as an adverse side effect of drugs used to treat UC (7).

Treatment 

The first line treatment consists in the use systemic corticosteroids, which usually helps in the remission of the oral manifestations. Immunosuppressive and biological agents are also commonly used.

-Celiac disease

Celiac disease (CD), also known as “Coeliac sprue”, is an autoimmune disease in which genetically predisposed individuals exhibit damages in the small intestine villi as a consequence of an abnormal immune response subsequent to the ingestion of gluten (7). The diagnosis of CD is made from the clinical and histological findings, which also allows to classify this disease into four main types; classical, atypical, silent and latent (19).

Etiopathogenia

Gluten (present in most cereals) is partially degraded by the action of gastro-intestinal enzymes into peptides that passes into the intestinal corion due to an increased permeability of the epithelial barrier. Once in the lamina propia, peptides are modified by tissue transglutaminase, which increases their affinity for receptors on the antigen presenting cells (APC’s) and thus their immunogenicity (20). The presentation of these peptides to CD4+ lymphocytes triggers an adaptive immune response with the consequent inflammation and tissue damage produced by the release of cytokines and matrix metalloproteinases (19).

Epidemiology

It has been estimated that CD affects approximately 1% of the world’s population (21), but over the last years, CD has experienced a large increase, affecting 1 in every 85 to 300 people (20). Celiac disease is more common in European countries as well as developing regions such as South America, South Asia and South Africa. Despite being typically developed in childhood, recent studies reported an increase in the involvement of adults. Women are considerably more affected than men, presenting a female-male ratio from 7:1 (21).

Oral Manifestations

Numerous authors have described different oral manifestations associated with CD, which are particularly important, considering that 50% of the patients with CD do not exhibit digestive symptoms at the time of diagnosis (28). Furthermore, oral lesions would be useful in early detection of atypical CD, which corresponds to the most common form of this disease (20).

Dental enamel defects: Patients with CD show increased risk for enamel developmental abnormalities, specifically enamel hypoplasia (22). In the temporal dentition the most affected teeth are second molars, while in the permanent teeth the central incisors are most commonly affected Generally, enamel hypoplasia is distributed bilaterally and symmetrically in both dental arches (20,22).

Atrophic glossitis and glossodynia: Even though depapilation and tongue burning sensation have been described as oral repercussions of CD, these manifestations are less common compared to other oral signs and symptoms (20). Despite the above, an analysis of 128 cases of CD cataloged the tongue as the most common affected oral site; 29.6% of the cases presented a painful-burning sensation and 8.6% erythema or atrophy of the tongue (7). Nevertheless, these signs and symptoms are likely to be secondary to anemia and hematinic deficiencies, often seen in celiac patients, rather than be caused by the disease itself (23).

Salivary flow and saliva composition: A decrease in salivary flow rates have been reported to be associated with the active phase of the disease, resulting in a dry mouth and burning sensation of the tongue (20).

Caries: Different studies (20,24) have described a higher rate of caries in celiac patients. This higher cariogenic risk would be supported by the increased susceptibility to caries of the hypoplastic enamel and the above mentioned alterations in salivary flow rates and saliva composition seen in CD patients.

RAS like oral ulceration: So far, there is no consensus on the relationship between aphthous ulcers and CD. Some researches (25) support the association between these two diseases, while others do not (26,27). It is likely that the appearance of RAS like ulcers is secondary to anemia and hematinic deficiencies (28).

Bleeding tendency: Celiac disease has been associated with alterations in coagulation, which would facilitate the onset of bleeding in the affected patients (e.g. epistaxis and skin bleeding). This coagulopathy is the result of abnormalities in prothrombin caused by poor absorption of vitamin K (29).

Dermatitis herpetiformis: Corresponds to a dermatological disorder strongly associated with celiac disease. In most of the cases the signs (mainly vesicles) and symptoms of itching, burning or pain affect the skin (e.g. buttocks, elbow), although some patients may experience lesions in the oral mucosa. The oral manifestations include erythematous-purpuric macules, erosions, ulcers and vesicles, which can affect the tongue, buccal mucosa and alveolar ridge (30). Clinically, it is difficult to differentiate it from other blistering diseases such as pemphigus or pemphigoid, so histological and immunofluorescence studies are necessary when suspected.

Treatment

The treatment of CD consists in the elimination of gluten from diet (e.g. cereals, such as wheat, rye and barley) which is effective in the remission of signs and symptoms, including those present in the oral cavity. This dietary restriction also applies in cases without gastrointestinal symptoms, because they play a protective role against long-term complications, such as the development of malignancies, specially non-Hodgkin´s lymphoma, in both gastrointestinal and extra intestinal sites (31).

-Gastroesophageal reflux disease

Gastroesophageal reflux (GER) is considered a normal physiological event of the human body (32). This natural process involves the regurgitation of gastric contents into the esophagus, which is then removed and neutralized by several protecting factors (e.g. esophageal peristalsis and saliva) (33). In some individuals this reflux of gastric and duodenal contents towards the esophagus generates a clinical picture called gastroesophageal reflux disease (GERD), characterized by the occurrence of different clinical signs and symptoms that are usually located in the esophagus (esophageal syndrome) (34) Other organs can also be affected, such as the pharynx, larynx, respiratory system and the oral cavity. When this happens, it is known as extra esophageal syndrome. GERD classical symptoms are heartburn and sour taste, but dysphagia, sore throat, odynophagia, globus sensation and nausea are also commonly reported (32-34).

Epidemiology

GERD is a global health problem with a high incidence and prevalence, and is becoming more common among children and adults. GERD usually manifests in the fourth decade of life with a predilection for females (32), and affects up to 20% of the Western European and North American population (34).

Etiopathogenia

Different mechanical barriers and physiological mechanisms, such as salivary flow rates, swallowing mechanisms and esophageal peristalsis, tend to keep in balance the GER. The impairment on these regulatory factors and an increase in the flow of stomach contents into the esophagus and superior aero digestive tract, can lead to the appearance of GERD 34).

Oral manifestations

Dental erosion: Is one of the most common extra-esophageal manifestations. Up to 44% of GERD patients present dental erosions within the course of the disease (35,36). It usually affects the lingual or palatal surface of the anterior teeth (37). The severity can be variable, with most cases showing only a mild loss of enamel, while others can have a severe exposure of dentin (35).

Xerostomia: It is likely that xerostomia appears as an adverse side effect of the medication taken for treating GERD, rather than being cause by GERD itself. Proton pump inhibitors are the first drug of choice and are likely to cause dry mouth sensation (37).

Halitosis: Even though the main determinants of halitosis or bad breath correspond to the patient’s oral conditions (e.g. periodontal problems or tongue coating), an increased risk of halitosis has been reported in in cases of symptomatic GERD. This was explained by a diminished function of the lower esophageal sphincter, which would facilitate flow of gases and gastric contents into the esophagus, producing the characteristic bad smell (38).

Mucositis: It might appear due the contact of the acids or its vapors with the oral mucosa. The oral mucosa is observed erythematous, generally on the palate and uvula, and the patient may complaint of burning sensation and/or pain (32). In some cases, the damage can be only microscopic, so no clinical signs can be seen (but the patient may still accuse some symptoms, such as burning sensation) (33).

Others: It has also been reported a higher incidence of RAS like ulceration, sour taste and burning mouth (37,38). RAS like ulcerations are likely to be secondary to anemia or hematinic deficiencies, which are not uncommon among these patients. Some patients may also experience an exaggerated sensitivity of the oral tissues to tactile stimuli (hyperesthesia) probably due to local irritation by the gastric reflux.

Treatment

The use of proton pump inhibitors is the treatment of choice. It provides the advantage of being non-invasive, cost effective and can serve as a diagnostic criterion for GERD. In some cases, drug therapy is not sufficient to control the symptoms, so an anti-reflux surgery can be considered (32).

## Conclusions

Although the frequency of oral manifestations is variable across GIDs and in most cases is non-specific (such as RAS like ulceration, stomatitis, burning sensation, etc.), these alterations may precede the underlying disease and therefore can facilitate an opportune diagnosis.
